# Lipid metabolism and the immune microenvironment in gastric cancer

**DOI:** 10.3389/fimmu.2026.1890119

**Published:** 2026-08-20

**Authors:** Zhuoyang Wang, Yichen Du, Qinglin Gu, Xinjie Liu, Ming Lu

**Affiliations:** 1 First Clinical Medical College, Anhui Medical University, Hefei, Anhui, China; 2 Second Clinical Medical College, Anhui Medical University, Hefei, Anhui, China; 3 Department of Immunology, School of Basic Medical Sciences, Anhui Medical University, Hefei, Anhui, China

**Keywords:** CD36, fatty acid oxidation, fatty acid synthase, gastric cancer, immunotherapy resistance, lipid metabolism, saturated and unsaturated fatty acids, sterol regulatory element-binding protein 1

## Abstract

The treatment of gastric cancer (GC) has entered an era of precision medicine combining molecular subtyping, immune checkpoint inhibitors (ICIs), anti-human epidermal growth factor receptor 2 (HER2), anti-claudin 18.2 (CLDN18.2), anti-angiogenic therapy, and chemotherapy. However, efficacy remains limited by tumor microenvironment (TME) heterogeneity, immune exclusion, myeloid suppression, nutrient competition, and metabolic adaptation. Lipid metabolic reprogramming represents a class of mechanisms with high translational value among metabolic immune checkpoints in GC: it supports tumor-cell membrane synthesis, redox homeostasis, peritoneal/omental metastasis, and adaptation to therapeutic stress, while also affecting regulatory T cells (Tregs), tumor-associated macrophages (TAMs), myeloid-derived suppressor cells (MDSCs), dendritic cells, and CD8+ T cells. This review focuses on cluster of differentiation 36 (CD36)-mediated fatty acid uptake; carnitine palmitoyltransferase 1A (CPT1A)-dependent fatty acid oxidation (FAO); fatty acid synthase (FASN), acetyl-CoA carboxylase (ACC), sterol regulatory element-binding protein 1 (SREBP-1), and stearoyl-CoA desaturase 1 (SCD1)-mediated *de novo* lipogenesis; cholesterol and lipid-droplet metabolism; lipid-mediated post-translational modifications; lipid peroxidation; and ferroptosis. Lipid metabolism-targeted therapy should be positioned as a biomarker-driven combination strategy rather than as non-selective monotherapy. Future studies should integrate single-cell and spatial transcriptomics with lipidomics, imaging mass spectrometry, and paired pre- and post-treatment biopsies to define cell type-specific lipid dependencies and pharmacodynamic markers. Clinical translation will require prospective validation that a candidate intervention changes the intended lipid pathway in the intended tumor or immune-cell compartment without disabling metabolically essential normal tissues.

## 1 Introduction: lipid metabolism in gastric cancer and the immune microenvironment

Gastric cancer remains a major digestive-system malignancy worldwide. Treatment of advanced or metastatic gastric/gastroesophageal junction adenocarcinoma has shifted from chemotherapy alone to precision combinations guided by human epidermal growth factor receptor 2 (HER2) status, programmed death-ligand 1 (PD-L1) combined positive score (CPS), microsatellite instability/mismatch repair (MSI/MMR) status, claudin 18.2 (CLDN18.2) expression, eligibility for anti-angiogenic therapy, and patient performance status (1–5). Nevertheless, programmed cell death protein 1 (PD-1)/PD-L1 blockade combined with chemotherapy does not overcome all forms of resistance; a considerable proportion of patients show primary non-response, transient benefit, or acquired progression.

The tumor microenvironment (TME) is a dynamic niche composed of tumor cells, immune cells, fibroblasts, vascular endothelial cells, extracellular matrix, and metabolites (6–9). Within this niche, tumor cells reshape immune-cell fate through nutrient competition and metabolite release. Metabolic immune checkpoints are functional nodes that connect metabolic pathways with immunosuppressive states (10–12). Compared with adenosine, lactate, and amino-acid pathways, lipid metabolism directly links GC-cell autonomy, peritoneal/omental metastasis, treatment resistance, and immune suppression. CD36, CPT1A/FAO, FASN/SREBP-1, SCD1, lipid droplets, and ferroptosis also provide measurable biomarkers and experimentally accessible targets (13–17). This review therefore focuses on lipid metabolism in GC and its relationship to the immune microenvironment, treatment resistance, and rational combination strategies.

### 1.1 Literature search strategy and evidence appraisal

PubMed, Web of Science, and Google Scholar were searched from database inception through 10 July 2026, and the reference lists of eligible primary studies and high-quality reviews were screened for additional records. Search concepts combined disease terms (“gastric cancer”, “gastric adenocarcinoma”, or “gastroesophageal junction adenocarcinoma”) with lipid-metabolism terms (including “fatty acid uptake”, “CD36”, “FAO”, “CPT1A”, “FASN”, “ACC”, “SREBP-1”, “SCD1”, “cholesterol”, “lipid droplet”, “palmitoylation”, “lipid peroxidation”, and “ferroptosis”) and immune or treatment terms (including “Treg”, “TAM”, “MDSC”, “dendritic cell”, “CD8 T cell”, “immune checkpoint inhibitor”, “chemotherapy resistance”, and “immunotherapy resistance”). Representative Boolean combinations included “gastric cancer AND CD36”, “gastric cancer AND SCD1”, and “lipid peroxidation AND CD8 T cell AND cancer”.

Eligible evidence included clinical cohorts, patient tissues, single-cell or spatial studies, organoids, patient-derived models, animal models, and mechanistic cell studies that addressed lipid metabolism, immune remodeling, metastasis, or treatment response in GC. Mechanistic studies from other solid tumors were included only when they clarified a biologically relevant GC hypothesis and are explicitly identified as cross-cancer evidence. Duplicate reports, non-peer-reviewed materials, studies with minimal relevance to GC immunometabolism, and purely predictive bioinformatic studies without mechanistic interpretation were excluded.

Evidence was appraised using four levels. Level 1 comprises GC or gastroesophageal junction patient samples, clinical cohorts, spatial or single-cell studies, paired treatment samples, and prospective clinical studies. Level 2 comprises GC cell lines, organoids, patient-derived models, animal models, and functional validation in GC tissues. Level 3 comprises mechanistic evidence from other solid tumors with strong biological relevance to the GC TME. Level 4 comprises testable hypotheses based on pathway biology, drug accessibility, and translational logic. **Tables 1** and **2** use these levels consistently.

**Table 1:** Evidence stratification and translational judgment for lipid metabolism and immune microenvironment in gastric cancer.

Lipid metabolism node	Main target cells	Core immune effect	Gastric cancer evidence level	Translational judgment
CD36 fatty acid uptake	GC cells, Tregs, TAMs, MDSCs, CD8^+^ T cells	Supports fatty acid utilization, Treg/TAM adaptation; may induce CD8^+^ T cell lipid peroxidation	Levels 1-3; strong for metastasis, immune mechanisms need GC validation (18, 21, 22, 26, 27)	Candidate core node; requires cell source and spatial localization validation
FAO/CPT1A	GC cells, Tregs, TAMs, MDSCs	Maintains oxidative metabolism, stress adaptation, and immunosuppressive cell function	Levels 2-4; supported by GI tumor models and reviews (29–31)	Suitable for combination exploration; needs attention to systemic toxicity
FASN/SREBP-1	Primarily GC cells	Promotes saturated-fatty-acid synthesis, MUFA production, membrane/lipid-droplet formation, stemness, metastasis, and adaptive signaling	Levels 1-2; direct GC tissue and mechanistic evidence (32, 33, 35, 37)	Good direct evidence; immune association needs strengthening
Cholesterol/oxysterols	Tumor cells, T cells, myeloid cells	Bidirectionally regulates TCR clustering, ER-stress-associated exhaustion, immune-cell migration, and myeloid recruitment	Levels 1 and 3; GC immune association plus cross-cancer mechanisms (41, 42, 44)	Emerging direction; not to be over-expanded
Lipid peroxidation/ferroptosis	Tumor cells, CD8^+^ T cells, myeloid cells	Tumor cell ferroptosis may be beneficial; CD8^+^ T cell ferroptosis may impair immunity	Levels 2-3; direct GC PUFA-ferroptosis evidence and cross-cancer immune evidence (26, 27, 36)	Must distinguish tumor cells from effector T cells
SFA/MUFA/PUFA balance	GC cells and immune cells	Controls membrane saturation, signaling, lipid storage, eicosanoid output, and ferroptosis sensitivity	Levels 1-3; direct GC palmitate, SCD1, lipidomic, and PUFA evidence (18, 36–38)	Named lipid species and cell source should be reported
Protein lipidation and bioactive lipid signaling	GC cells, T cells, myeloid cells, ILC3s	Stabilizes immune-checkpoint proteins or transmits PGE2, oxysterol, and LysoPS signals	Levels 1 and 3; direct GC LysoPS evidence and cross-cancer palmitoylation/PGE2 evidence (40, 45, 48)	Promising mechanistic bridge; requires GC-specific validation for most PTMs

**Table 2:** Directions, risks, and biomarkers of lipid metabolism-targeted therapy.

Therapeutic direction	Representative strategies	Current evidence	Main risks	Suggested biomarkers/combination
CD36-mediated uptake	SSO; research anti-CD36 antibodies	Preclinical; direct GC target biology, no GC clinical efficacy (18, 28, 43)	Systemic lipid handling and macrophage scavenging	Tumor/immune-cell CD36 localization, uptake assay, palmitate/oxLDL signature
CPT1A/FAO	Etomoxir; perhexiline; ranolazine (repurposing concept)	GI tumor and cross-cancer preclinical evidence (30, 31)	Cardiac, hepatic, neuromuscular, and memory-T-cell toxicity; off-target effects	CPT1A/FAO flux, acyl-carnitines; short or sequential combination with chemotherapy/ICI
ACC/FASN/SREBP-1/SCD1	ND-646; C75/orlistat; TVB-2640 (denifanstat); fatostatin/PF-429242; A939572; Penicolinate H	Direct GC mechanisms for SREBP-1/SCD1/Penicolinate H; early solid-tumor trial for TVB-2640 (33, 35, 37, 47)	Skin/ocular and systemic lipid effects; compensatory CD36 uptake (34, 47)	FASN/SREBP-1/SCD1 expression, isotope-traced lipogenesis, MUFA: SFA ratio, lipid droplets
Cholesterol esterification/signaling	Avasimibe (ACAT/SOAT inhibitor)	Cross-cancer preclinical enhancement of TCR signaling and anti-PD-1 response (42)	Opposite effects of membrane versus ER cholesterol; systemic dyslipidemia	Cell-compartment cholesterol, TCR clustering, XBP1/ER-stress signature
PGE2 and related lipid mediators	Celecoxib; TPST-1495 (dual EP2/EP4 antagonist)	Cross-cancer mechanistic/preclinical evidence; direct GC pathway relevance requires validation (45, 48)	Cardiovascular, gastrointestinal, and pathway-redundancy concerns	PGE2, EP2/EP4, COX-2/mPGES-1; combine with ICI in biomarker-positive tumors
Ferroptosis modulation	Erastin/RSL3 to induce; ferrostatin-1/liproxstatin-1 to suppress	Direct GC PUFA-ferroptosis biology plus cross-cancer CD8+ T-cell mechanisms (26, 27, 36)	Tumor-cell and CD8+ T-cell effects may be opposite	Cell type-specific ACSL4/GPX4/SLC7A11 score, oxidized phospholipids, CD8+ function

## 2 Lipid metabolism in gastric cancer

Lipid metabolic reprogramming includes exogenous fatty acid uptake, *de novo* fatty acid synthesis, FAO, phospholipid and sphingolipid remodeling, cholesterol metabolism, lipid-droplet formation, lipid signaling, lipid-mediated protein modifications, lipid peroxidation, and ferroptosis. Compared with glucose or amino-acid metabolism, these pathways are tightly connected to membrane architecture, signaling platforms, energy storage, redox homeostasis, and cell-fate control; importantly, the same lipid pathway can produce opposite consequences in tumor cells and different immune-cell subsets (13–15, 17).

Lipid class and degree of unsaturation are mechanistically important. Saturated fatty acids (SFAs), exemplified by palmitate (16:0) and stearate (18:0), can increase membrane saturation and lipotoxic or inflammatory stress; in GC, palmitate also activates the CD36-AKT/GSK-3beta/beta-catenin metastatic axis (18). SCD1 converts saturated acyl-CoAs into monounsaturated fatty acids (MUFAs), particularly palmitoleate (16:1) and oleate (18:1), thereby supporting membrane fluidity, neutral-lipid storage, stemness, and metastatic fitness (37). Polyunsaturated fatty acids (PUFAs), including linoleic, arachidonic, and adrenic acids, are substrates for eicosanoid synthesis and phospholipid peroxidation; the ELOVL5/FADS1 pathway determines ferroptosis sensitivity in GC (36). Glycerophospholipids, sphingomyelins, triacylglycerols, cholesterol/oxysterols, and lysophospholipids are also altered in GC and can function as structural lipids, circulating biomarkers, or immune signals (38, 45, 49).

At the tumor-cell level, lipid metabolism supports proliferation, membrane synthesis, signal transduction, epithelial-mesenchymal transition (EMT), invasion, peritoneal dissemination, and treatment tolerance. Peritoneal and omental metastases are closely associated with adipose tissue, from which GC cells can acquire exogenous fatty acids. Adipocytes support omental metastasis through a phosphatidylinositol transfer protein cytoplasmic 1 (PITPNC1)-related mechanism (16), whereas palmitate promotes metastatic phenotypes through the CD36-AKT/glycogen synthase kinase 3 beta (GSK-3beta)/beta-catenin axis (18).

At the immune-cell level, lipid metabolism shapes the relative fitness of immune subsets in the TME. Recently activated CD8+ effector T cells require high glycolytic flux to support proliferation, cytokine production, and cytolysis, whereas Tregs, M2-like TAMs, and subsets of MDSCs can more readily use fatty acids and oxidative metabolism in low-glucose, high-lactate, hypoxic environments (12, 19–22). Lipid-related impairment of effector metabolism is not a single switch: glucose depletion and lactate reduce glycolytic substrate availability; CD36-mediated uptake of oxidized low-density lipoprotein (oxLDL) drives phospholipid peroxidation and mitochondrial damage; and excess long-chain or very-long-chain fatty acids can accumulate when beta-oxidation capacity is inadequate, causing lipotoxic stress, loss of mitochondrial reserve, and reduced metabolic flexibility (26, 27, 39). Persistent cholesterol loading can additionally activate endoplasmic-reticulum stress and X-box binding protein 1 (XBP1), inducing inhibitory receptors and CD8+ T-cell exhaustion (41). These processes collectively weaken glycolysis-dependent effector function while favoring immune populations that tolerate or exploit lipid-rich conditions.

At the spatial-niche level, lipid-rich regions may overlap with hypoxia, TAM/MDSC enrichment, and CD8+ T-cell exclusion. Bulk expression of CD36, FASN, CPT1A, or SCD1 cannot identify which cells carry the relevant dependency. Spatially resolved studies should therefore co-map named lipid species (such as palmitate, oleate, arachidonic acid, oxysterols, and oxidized phospholipids), pathway proteins, immune-cell identity, and functional readouts, and should test whether these features correlate with Treg/TAM accumulation, CD8+ T-cell dysfunction, and poor ICI response (38, 44).

### 2.1 CD36-mediated fatty acid uptake: from metastasis to immunosuppressive niche

**Figure 1:**
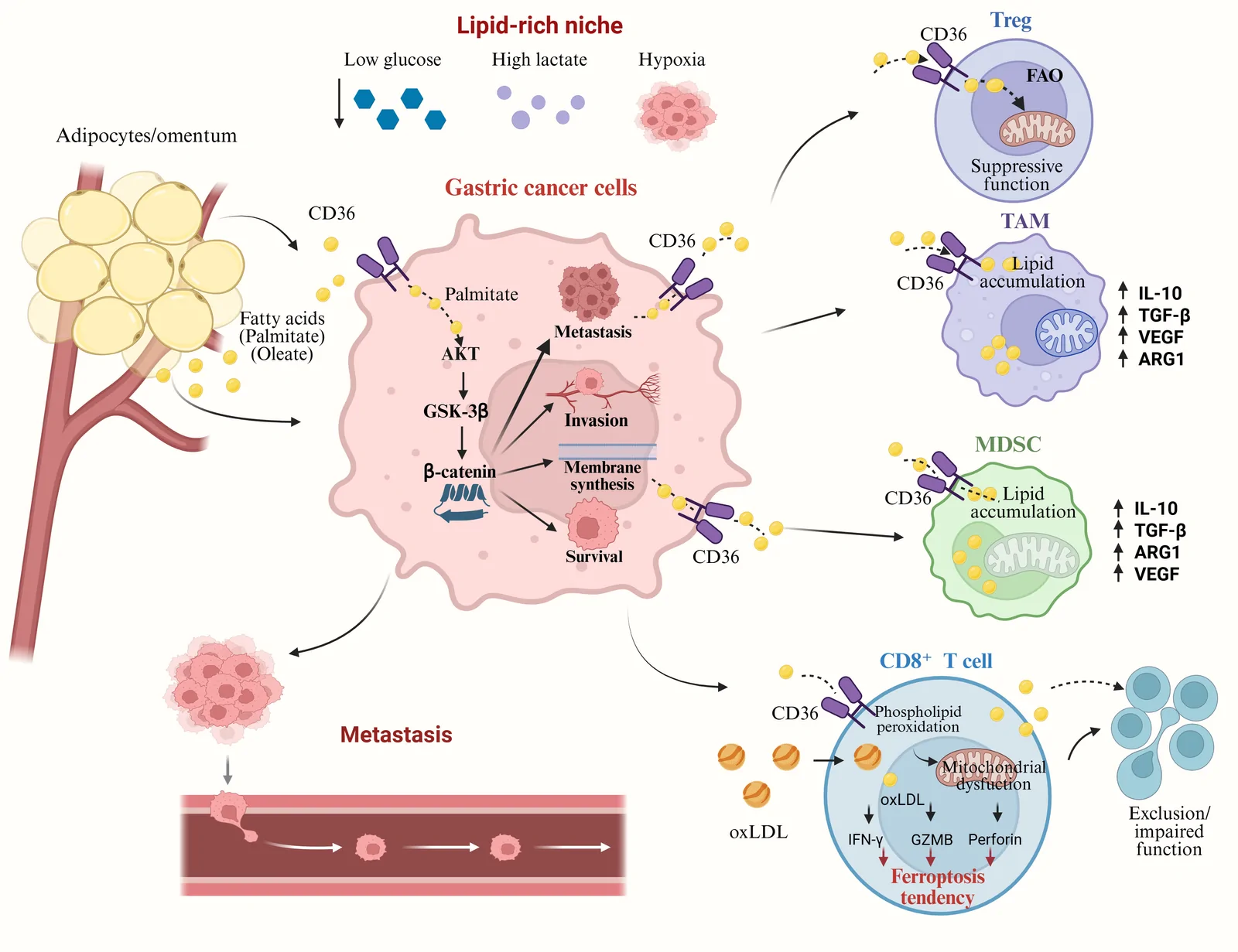
CD36-mediated fatty acid uptake in gastric cancer. Albumin-bound or adipocyte-derived long-chain fatty acids, including palmitate and oleate, are taken up by GC cells through CD36. Palmitate activates the AKT/GSK-3beta/beta-catenin pathway and promotes membrane synthesis, invasion, survival, and metastasis. In the lipid-rich TME, CD36 also supports Treg, TAM, and MDSC adaptation, whereas oxLDL uptake in CD8+ T cells induces phospholipid peroxidation, mitochondrial dysfunction, ferroptosis susceptibility, and reduced antitumor function.

Cluster of differentiation 36 (CD36) is a transmembrane fatty acid translocase and class B scavenger receptor that binds long-chain fatty acids, oxLDL, and other lipid ligands (**Figure 1**). In GC cells, CD36 supports uptake of exogenous fatty acids for mitochondrial oxidation, membrane synthesis, and signaling. In immune cells, the same receptor can support Treg survival, lipid accumulation in TAMs/MDSCs, and oxidized-lipid uptake followed by peroxidative injury in CD8+ T cells (23–27). Thus, the biological outcome depends on the lipid species, carrier, cell type, and downstream metabolic capacity rather than on CD36 abundance alone.

Direct evidence for CD36 in GC is strongest for the adipose-rich peritoneal and omental niche. The available extracellular pool includes albumin-bound non-esterified fatty acids released from adipose tissue, fatty acids liberated from circulating chylomicron or very-low-density lipoprotein triglycerides by lipoprotein lipase, and LDL/oxLDL particles; local adipocytes additionally supply palmitate and oleate. CD36 primarily facilitates uptake of released long-chain fatty acids and modified lipoprotein lipids rather than intact triglycerides. In GC models, palmitate activates the CD36-AKT/GSK-3beta/beta-catenin pathway (18), and hypoxia increases CD36 expression and fatty-acid-dependent peritoneal metastasis (43). Evidence remains insufficient to assign equal causal weight to every circulating fatty acid species. Future studies should pair plasma and ascites lipidomics with isotope tracing, lipoprotein profiling, and cell type-specific CD36 perturbation across primary, nodal, peritoneal, and omental lesions (16, 18, 38, 43, 49).

At the immune level, CD36-mediated fatty acid uptake helps Tregs maintain suppressive function in low-glucose, high-lactate, lipid-rich conditions (20, 22). Lipid accumulation in TAMs and MDSCs can reinforce interleukin 10 (IL-10), transforming growth factor beta (TGF-beta), vascular endothelial growth factor (VEGF), arginase 1 (ARG1), and PD-L1-associated suppressive programs (21). In CD8+ T cells, CD36-mediated oxLDL uptake promotes lipid peroxidation, mitochondrial dysfunction, ferroptosis susceptibility, and loss of interferon gamma (IFN-gamma), granzyme B, and perforin (26, 27). These immune mechanisms are compelling but are still supported mainly by cross-cancer models and require validation in paired GC samples collected before and after ICI treatment.

Because CD36 also participates in intestinal lipid handling, macrophage scavenging, angiogenic signaling, and systemic metabolic homeostasis, its expression alone is not a sufficient therapeutic biomarker. Translational studies should distinguish CD36 on tumor cells from CD36 on Tregs, TAMs, MDSCs, endothelial cells, and CD8+ T cells; quantify pathway activity with lipid-uptake and peroxidation assays; and define whether the dominant biological effect is tumor fueling, myeloid/Treg adaptation, or effector T-cell injury.

### 2.2 FAO/CPT1A: metabolic adaptation of immunosuppressive cells and therapeutic window

**Figure 2:**
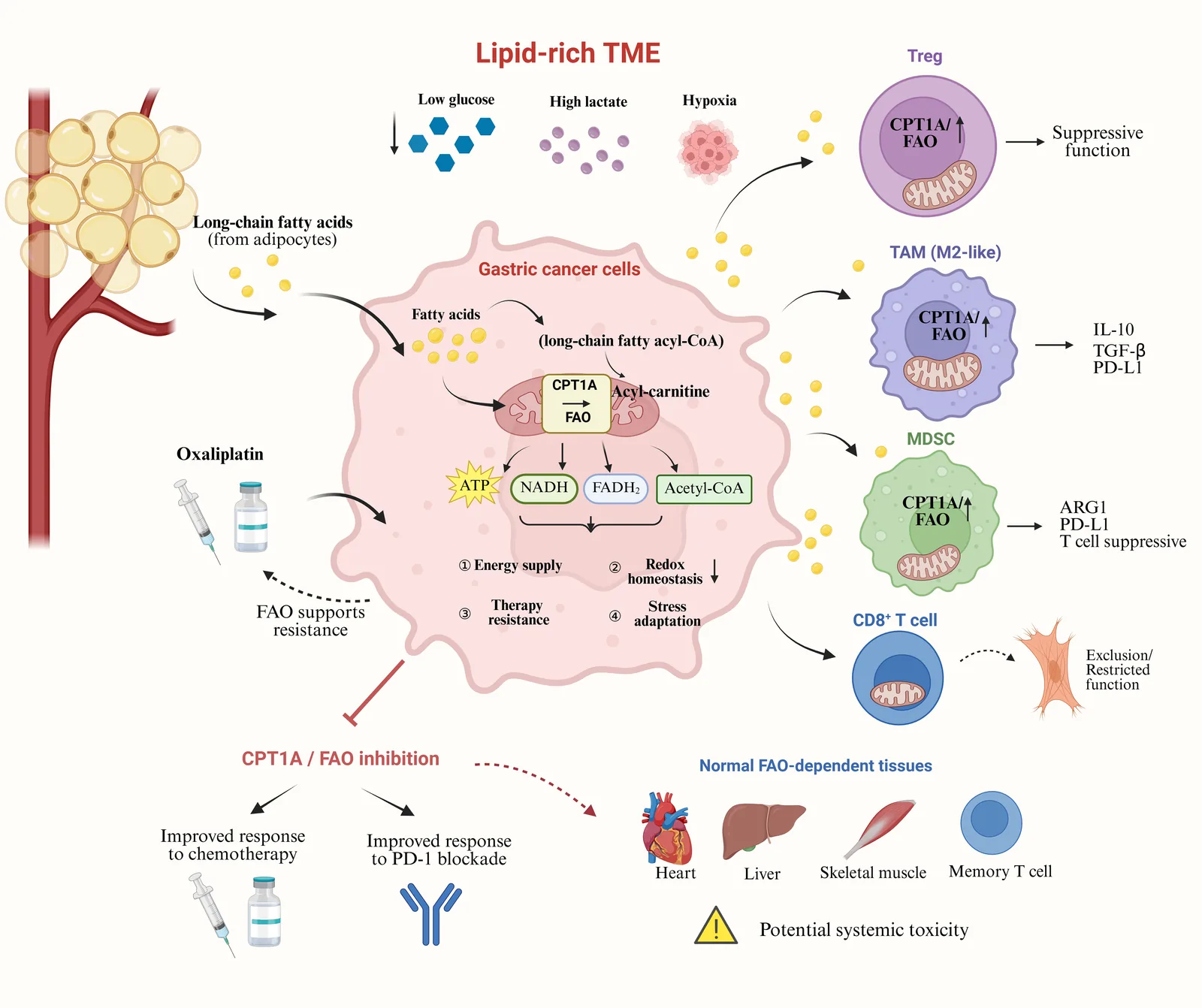
CPT1A/FAO-mediated metabolic adaptation. CPT1A converts long-chain fatty acyl-CoA to acyl-carnitine for mitochondrial entry and beta-oxidation, generating acetyl-CoA, NADH, FADH2, and ATP. This process supports GC-cell redox homeostasis, stress adaptation, and chemotherapy resistance and can favor Treg, M2-like TAM, and MDSC suppressive programs. The figure also indicates the potential toxicity of systemic FAO inhibition in the heart, liver, skeletal muscle, and memory T cells.

Fatty acid oxidation (FAO) is the mitochondrial process that converts fatty acyl-CoA into acetyl-CoA, NADH, and FADH2. Carnitine palmitoyltransferase 1A (CPT1A), located on the outer mitochondrial membrane, converts long-chain fatty acyl-CoA to acyl-carnitine and controls its entry into mitochondria for beta-oxidation (**Figure 2**). FAO can support ATP production, redox balance, and stress adaptation in tumor cells; in immune cells it contributes to Treg maintenance, memory T-cell formation, M2-like macrophage polarization, and MDSC suppressive function (29). The relevant question is therefore which cell type uses FAO, under which nutrient conditions, and for which functional program.

Studies in gastrointestinal tumors have shown that inhibiting fatty acid catabolism enhances the efficacy of oxaliplatin-based chemotherapy (30); recent reviews have also identified FAO targeting as a potential strategy for gastrointestinal tumors (31). In GC, FAO activation may help tumor cells maintain energy and redox balance, thereby reducing the pressure from chemotherapy or immunotherapy. If FAO simultaneously supports Tregs, TAMs, or MDSCs, its significance would extend from “enhancing chemosensitivity” to “remodeling the immune microenvironment”.

The dependence of Tregs, TAMs, and MDSCs on lipid utilization makes CPT1A/FAO an important candidate node in the immunosuppressive niche. If the GC TME is in a low-glucose, high-lactate, lipid-rich state, it may selectively reinforce the advantage of Tregs and myeloid cells. Future studies could focus on verifying whether CPT1A^+^ Tregs/TAMs/MDSCs are enriched in CD8^+^ T cell-excluded areas and co-express ARG1, IL-10, TGF-β, PD-L1, or CD73.

FAO dependence also creates a biological trade-off: the heart, liver, skeletal muscle, and memory T cells require oxidative lipid metabolism, and systemic CPT1A/FAO suppression may impair these tissues. In addition, tumor-infiltrating CD8+ T cells can need FAO to maintain energy under nutrient stress, while persistent ACC activity can divert fatty acids toward synthesis and storage rather than oxidation (46). These cell type-specific effects define the therapeutic window and should be measured directly rather than inferred from bulk CPT1A expression.

### 2.3 FASN/SREBP-1 and *de novo* fatty acid synthesis

**Figure 3:**
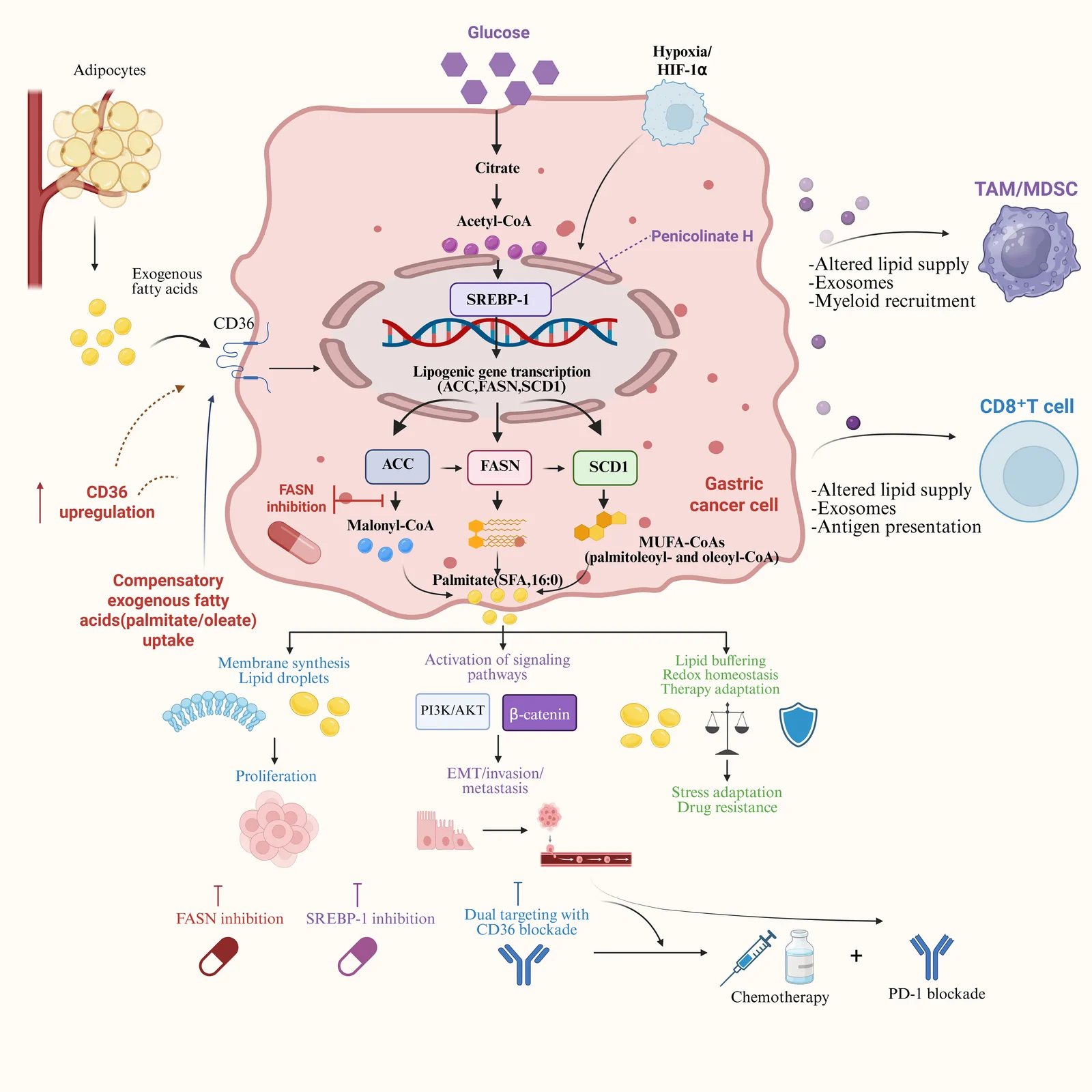
FASN/SREBP-1-driven *de novo* lipogenesis. The SREBP-1-ACC-FASN-SCD1 axis drives *de novo* lipogenesis in GC. ACC generates malonyl-CoA, FASN synthesizes palmitate, and SCD1 converts palmitoyl-CoA and stearoyl-CoA into Monounsaturated fatty acyl-CoAs (MUFA-CoAs), principally palmitoleoyl-CoA and oleoyl-CoA. These named lipid products support membrane synthesis, lipid droplets, proliferation, EMT/invasion, redox balance, and drug resistance. FASN inhibition may induce compensatory CD36 upregulation, supporting evaluation of combined lipogenesis and uptake blockade.

In addition to exogenous fatty acid uptake, GC cells can meet proliferative and metastatic demands through *de novo* lipogenesis (**Figure 3**). Acetyl-CoA carboxylase (ACC, particularly cytosolic ACC1) converts acetyl-CoA to malonyl-CoA, the committed substrate for fatty acid synthesis. Fatty acid synthase (FASN) uses acetyl-CoA, malonyl-CoA, and NADPH to generate palmitate. Sterol regulatory element-binding protein 1 (SREBP-1) is an endoplasmic-reticulum-bound transcription factor that, after regulated processing, induces genes including ACLY, ACC, FASN, and SCD1. Stearoyl-CoA desaturase 1 (SCD1) converts palmitoyl-CoA and stearoyl-CoA into the monounsaturated products palmitoleoyl-CoA and oleoyl-CoA, increasing membrane fluidity and lipid-droplet compatibility. GC studies link FASN with hypoxia-inducible factor 1 alpha (HIF-1alpha)/SREBP-1c and show that SREBP-1c and SCD1 promote lipogenesis, stemness, growth, and metastasis (32, 33, 37).

The FASN/SREBP-1/SCD1 program supports tumor adaptation by supplying membrane phospholipids and neutral-lipid stores; remodeling signaling platforms that engage phosphoinositide 3-kinase (PI3K)/AKT, beta-catenin, Hippo/yes-associated protein (YAP), and EMT pathways; and buffering saturated-fatty-acid toxicity and oxidative stress through monounsaturated fatty acids and lipid droplets (32, 33, 37). Its immune effects can be direct or paracrine: altered phospholipid, exosome, oxysterol, and eicosanoid output can influence antigen presentation, myeloid recruitment, and immune-receptor signaling. These immune links should be tested with co-culture and spatial lipidomic approaches rather than inferred from tumor-cell lipogenic gene expression alone.

Metabolic plasticity connects *de novo* synthesis with exogenous uptake. Experimental FASN suppression can increase CD36 expression and redirect cells toward extracellular fatty acids (34), whereas SCD1-dependent desaturation determines whether newly synthesized saturated fatty acids are incorporated into membranes, stored, or become lipotoxic. Consequently, a high FASN/SREBP-1 signature and a high CD36 uptake signature may identify distinct or coexisting dependencies rather than interchangeable biomarkers.

Direct GC studies support the existence of lipogenesis-dependent states: SREBP-1c activation promotes growth and metastasis (33), SCD1 supports GC stem-like properties and chemotherapy resistance through Hippo/YAP signaling (37), and the marine-derived compound Penicolinate H exposes an SREBP-1-mediated vulnerability (35). Future classification should test whether these states associate with HER2, CLDN18.2, MSI, Epstein-Barr virus (EBV), Lauren subtype, or peritoneal-metastatic status.

### 2.4 Cholesterol metabolism, lipid droplets, and lipid peroxidation

**Figure 4:**
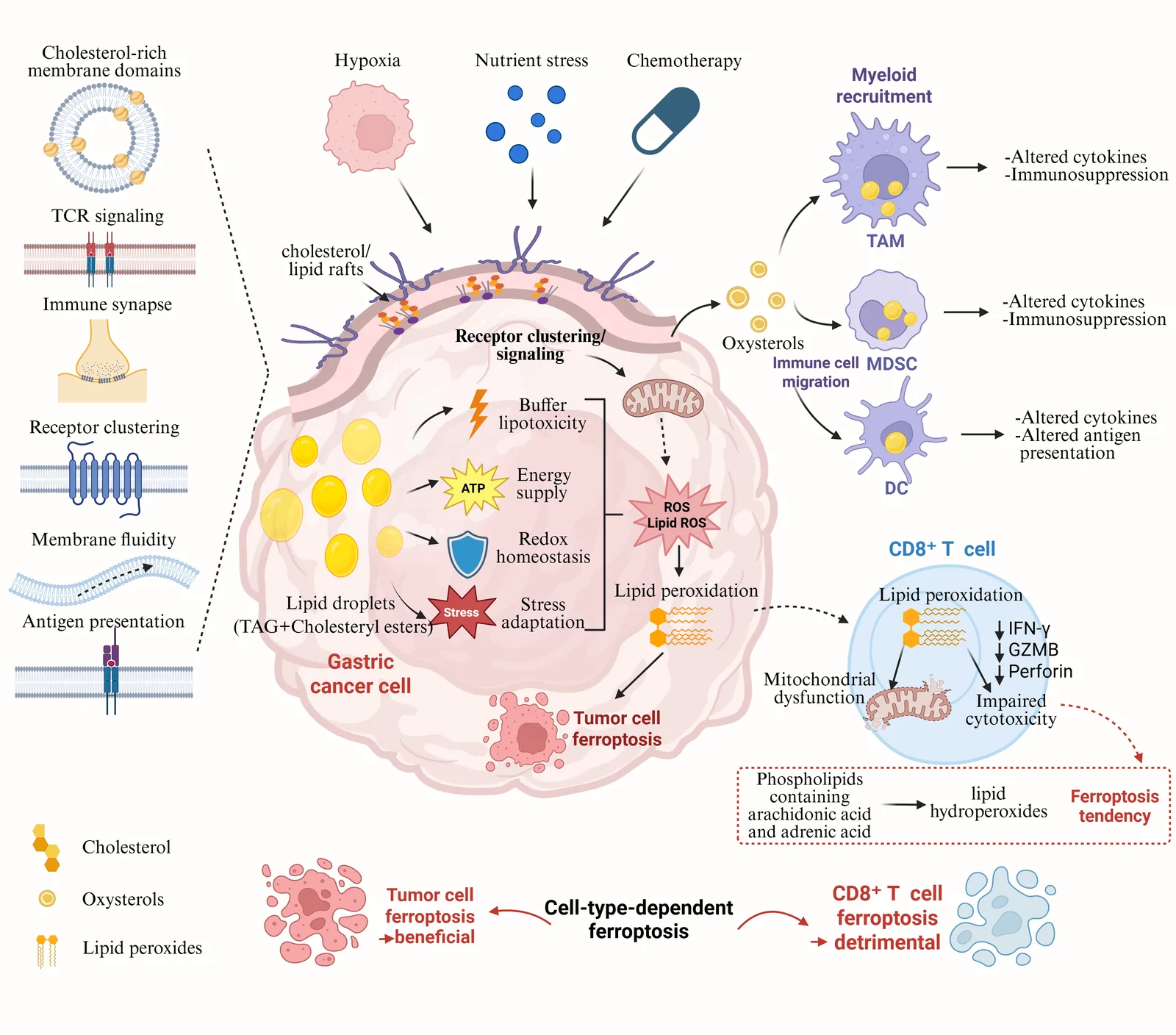
Cholesterol, lipid droplets, lipid peroxidation, and ferroptosis. Cholesterol and cholesteryl esters regulate receptor clustering, immune-synapse formation, TCR signaling, and membrane fluidity; oxysterols act as immune-migration signals. Lipid droplets store triacylglycerols and cholesteryl esters and buffer metabolic stress. Arachidonic- and adrenic-acid-containing phospholipids are oxidized to lipid hydroperoxides during ferroptosis. Tumor-cell ferroptosis may be beneficial, whereas excessive phospholipid peroxidation in CD8+ T cells impairs cytotoxicity.

Cholesterol is a structural component of membranes and lipid rafts that controls T-cell receptor (TCR) clustering, immune-synapse formation, membrane fluidity, receptor trafficking, and antigen presentation (**Figure 4**). Its effects are bidirectional and compartment-specific. Increased plasma-membrane cholesterol can strengthen TCR signaling, whereas excess intracellular cholesterol can trigger endoplasmic-reticulum stress/XBP1 and inhibitory-receptor expression in CD8+ T cells (41, 42). Tumor-derived oxysterols can also influence liver X receptor signaling and myeloid-cell migration. In GC, serum lipoprotein-cholesterol states correlate with tertiary lymphoid structure and immune-cell features, but causal evidence for cholesterol-directed immunotherapy remains limited (44).

Lipid droplets are not passive depots; they store triacylglycerols and cholesteryl esters, buffer saturated-fatty-acid toxicity, regulate substrate delivery to mitochondria, and organize stress signaling. Under hypoxia, nutrient fluctuation, chemotherapy, or redox imbalance, GC cells can sequester fatty acids in droplets and later mobilize them for energy or membrane repair. In TAMs and MDSCs, lipid accumulation can support suppressive cytokine programs, whereas excessive lipid storage in dendritic cells may impair antigen processing and cross-presentation. The lipid species within droplets, not droplet number alone, should therefore be quantified.

Lipid peroxidation and ferroptosis require explicit cell-type and lipid-species resolution. Long-chain acyl-CoA synthetase 4 (ACSL4) enriches arachidonic- and adrenic-acid-containing phospholipids that are susceptible to oxidation, whereas glutathione peroxidase 4 (GPX4) reduces phospholipid hydroperoxides using glutathione supported by the cystine/glutamate antiporter subunit solute carrier family 7 member 11 (SLC7A11). In GC, the ELOVL5/FADS1 polyunsaturated-fatty-acid pathway determines ferroptosis sensitivity (36). Tumor-cell ferroptosis may enhance treatment sensitivity, but lipid peroxidation in CD8+ T cells damages mitochondria and reduces IFN-gamma, granzyme B, and perforin (26, 27). Ferroptosis should therefore never be labeled uniformly pro- or anti-tumor.

### 2.5 Lipid-dependent post-translational modifications and immune signaling

Lipid-derived covalent post-translational modifications provide a direct bridge between metabolism and signaling. Reversible S-palmitoylation attaches palmitate to cysteine residues, whereas N-myristoylation and prenylation add lipid groups that control protein-membrane recruitment, trafficking, stability, and assembly of signaling complexes. A mechanistically relevant example is PD-L1 S-palmitoylation by zinc finger DHHC-type palmitoyltransferase 3 (ZDHHC3), which limits PD-L1 ubiquitination and lysosomal degradation, thereby maintaining an immune-evasive protein pool; pharmacologic or genetic interruption restored T-cell activity in preclinical models (40). This mechanism has not yet been validated specifically in GC and is therefore classified as Level 3 evidence.

Non-covalent bioactive lipids also reshape tumor-immune signaling. PGE2 signals through EP2/EP4-cyclic adenosine monophosphate pathways to suppress CD8+ T cells and myeloid-cell activation (48); oxysterols alter liver X receptor-dependent migration and cholesterol homeostasis; and, in a direct GC study, lysophosphatidylserine (LysoPS) produced through ABHD16A activated group 3 innate lymphoid cells through GPR34-AKT-STAT3, induced IL-22, and increased tumor-cell PD-L1 expression (45). Together, protein lipidation and secreted lipid mediators explain how lipid metabolism can remodel the TME without serving primarily as an energy source.

## 3 Biomarker-driven therapeutic strategies and combination framework

### 3.1 Integrated targeting and combination strategies

**Figure 5:**
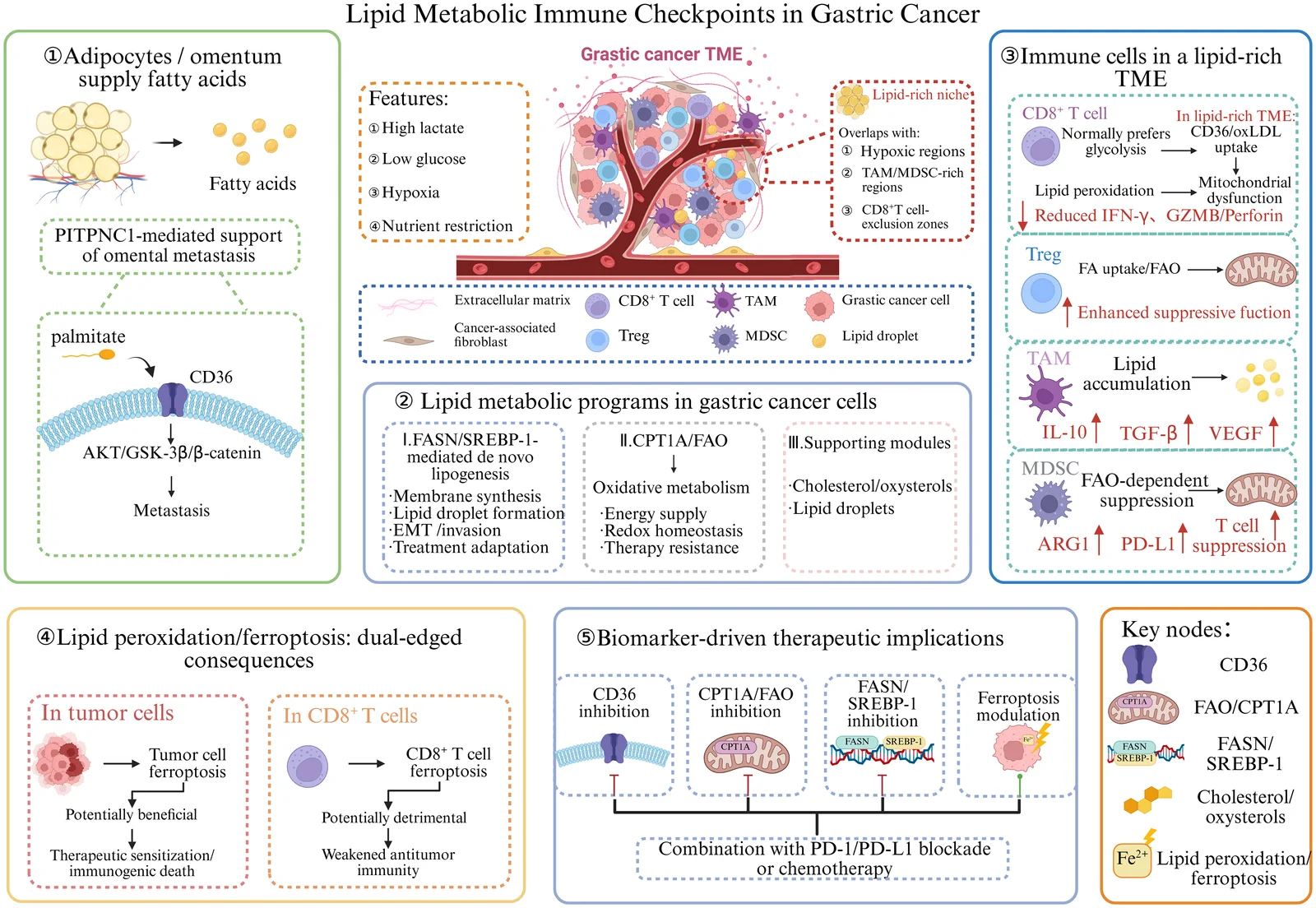
Integrated lipid metabolic immune checkpoints in gastric cancer. The summary model integrates adipocyte-derived fatty acids; CD36-mediated uptake; CPT1A/FAO; the FASN/SREBP-1/SCD1 lipogenic program; cholesterol, oxysterols, and lipid droplets; and cell type-dependent lipid peroxidation/ferroptosis. Therapeutic concepts are shown as biomarker-driven combinations with chemotherapy or PD-1/PD-L1 blockade rather than established GC treatments.

Candidate interventions fall into six mechanistic groups: inhibition of fatty acid uptake; modulation of CPT1A/FAO; inhibition of ACC/FASN/SREBP-1/SCD1-dependent lipogenesis; modulation of cholesterol esterification or lipid-raft signaling; blockade of immunosuppressive lipid mediators; and cell type-selective control of lipid peroxidation/ferroptosis (**Figure 5**). These categories are supported by different evidence levels, ranging from direct GC models to pan-cancer mechanistic studies and early solid-tumor trials (13–15, 17, 18, 28–35, 37, 47). None should be considered established standard treatment for GC.

Representative agents should be named together with their limitations. Sulfo-N-succinimidyl oleate (SSO) is a research inhibitor of CD36-associated fatty acid uptake (28). Etomoxir and perhexiline inhibit CPT1-dependent FAO but have important off-target or systemic-toxicity concerns and remain investigational in oncology (30, 31). Lipogenesis-directed tools include the FASN inhibitors C75, orlistat, and TVB-2640/denifanstat; the ACC inhibitor ND-646; the SREBP-processing inhibitors fatostatin and PF-429242; the SCD1 inhibitor A939572; and Penicolinate H (35, 37, 47). The ACAT/SOAT inhibitor avasimibe can increase plasma-membrane cholesterol and TCR clustering in CD8+ T cells in preclinical models (42). For immunosuppressive prostaglandin E2 (PGE2) signaling, celecoxib and the dual EP2/EP4 antagonist TPST-1495 illustrate pathway-level approaches (48). Erastin and RSL3 induce ferroptosis in experimental systems, whereas ferrostatin-1 and liproxstatin-1 suppress lipid peroxidation; these remain preclinical tools rather than GC therapies.

Combination design should be driven by mechanism and compartment. With PD-1/PD-L1 blockade, a lipid intervention should demonstrably increase CD8+ T-cell infiltration or function, reduce Treg/TAM/MDSC suppression, stabilize immune-receptor signaling, or prevent effector-cell lipid peroxidation (20–27, 40–42, 45, 48). With chemotherapy, inhibition of FAO, FASN/SREBP-1, or SCD1 may reduce redox buffering, lipid-droplet reserves, and membrane repair, thereby enhancing oxaliplatin- or taxane-associated stress (30, 35, 37, 47). Proposed combinations with anti-HER2, anti-CLDN18.2, or anti-VEGF/VEGFR therapy remain Level 4 hypotheses until direct GC data demonstrate effects on receptor organization, antibody-dependent cellular cytotoxicity, vascular lipid delivery, or immune-cell function.

Safety and biomarker selection are inseparable. CD36 blockade may disrupt physiological lipid handling and macrophage scavenging; FAO inhibition may injure heart, liver, skeletal muscle, or memory T cells; FASN/SREBP-1/SCD1 inhibition can trigger compensatory uptake; cholesterol manipulation can produce opposite effects in different compartments; and ferroptosis induction may damage CD8+ T cells while killing tumor cells. Trials should therefore require cell type-resolved target expression, a functional lipid-uptake or pathway signature, on-treatment pharmacodynamic sampling, and prespecified immune readouts. **Table 2** integrates representative agents, evidence, risks, and candidate biomarkers.

## 4 Conclusion

Lipid metabolism provides a mechanistic framework connecting GC growth, peritoneal/omental metastasis, treatment adaptation, myeloid suppression, and CD8+ T-cell dysfunction. The evidence synthesis distinguishes lipid classes and compartments: palmitate-driven CD36 signaling can promote GC metastasis; SCD1-derived monounsaturated fatty acids support membrane and stemness programs; arachidonic- and adrenic-acid-containing phospholipids determine ferroptosis sensitivity; cholesterol can either reinforce TCR clustering or induce stress-associated exhaustion; and lipid mediators or lipidation events can stabilize immunosuppressive signaling (16, 18, 36–45).

The evidence is uneven. Direct GC data support adipocyte-derived fatty-acid fueling, palmitate/CD36-dependent metastasis, hypoxia-induced CD36 expression, FASN/SREBP-1/SCD1-dependent progression, PUFA-regulated ferroptosis, serum lipidomic heterogeneity, and a LysoPS-ILC3-PD-L1 signaling axis (16, 18, 32, 33, 35–38, 43–45). By contrast, CD36-driven ferroptosis in CD8+ T cells, cholesterol-XBP1 exhaustion, PD-L1 palmitoylation, and several lipid-directed combinations are currently extrapolated from other tumor systems (26, 27, 40–42, 48). This distinction should guide the strength of translational claims.

Clinical translation should proceed in stages. First, retrospective and prospective GC cohorts should use spatial transcriptomics, multiplex imaging, lipidomics, and imaging mass spectrometry to define reproducible, cell type-specific lipid niches and link them to treatment response. Second, organoid-immune co-cultures, patient-derived models, and isotope tracing should verify that the proposed biomarker represents pathway dependence rather than correlation. Third, window-of-opportunity or early-phase combination trials should include paired biopsies, circulating and ascites lipid profiles, target-engagement assays, CD8+ T-cell functional readouts, and organ-specific safety monitoring. Only interventions that show a favorable therapeutic window and a measurable pharmacodynamic effect in the intended cell compartment should advance to biomarker-enriched efficacy testing. This roadmap positions lipid metabolism as a testable precision-immunotherapy strategy while avoiding premature claims of clinical readiness.

## Glossary

ABHD16A: Alpha/beta hydrolase domain-containing protein 16A
ACC: Acetyl-CoA carboxylase
ACSL4: Long-chain acyl-CoA synthetase 4
AKT: Protein kinase B
ARG1: Arginase 1
CD36: Cluster of differentiation 36
CLDN18.2: Claudin 18.2
CPS: Combined positive score
CPT1A: Carnitine palmitoyltransferase 1A
EBV: Epstein-Barr virus
EMT: Epithelial-mesenchymal transition
FAO: Fatty acid oxidation
FASN: Fatty acid synthase
GC: Gastric cancer
GEJ: Gastroesophageal junction
GPX4: Glutathione peroxidase 4
GSK-3beta: Glycogen synthase kinase 3 beta
HER2: Human epidermal growth factor receptor 2
HIF-1alpha: Hypoxia-inducible factor 1 alpha
ICI: Immune checkpoint inhibitor
IFN-gamma: Interferon gamma
IL: Interleukin
ILC3: Group 3 innate lymphoid cell
LysoPS: Lysophosphatidylserine
MDSC: Myeloid-derived suppressor cell
MMR: Mismatch repair
MSI: Microsatellite instability
MUFA: Monounsaturated fatty acid
NK: Natural killer
oxLDL: Oxidized low-density lipoprotein
PD-1: Programmed cell death protein 1
PD-L1: Programmed death-ligand 1
PGE2: Prostaglandin E2
PI3K: Phosphoinositide 3-kinase
PUFA: Polyunsaturated fatty acid
SCD1: Stearoyl-CoA desaturase 1
SFA: Saturated fatty acid
SLC7A11: Solute carrier family 7 member 11
SREBP-1: Sterol regulatory element-binding protein 1
TAM: Tumor-associated macrophage
TCR: T-cell receptor
TGF-beta: Transforming growth factor beta
TME: Tumor microenvironment
Treg: Regulatory T cell
VEGF: Vascular endothelial growth factor
XBP1: X-box binding protein 1
YAP: Yes-associated protein
ZDHHC3: Zinc finger DHHC-type palmitoyltransferase 3
